# Supplementary material for: Are carbon emissions trading and green financial instruments synergistic? -Comprehensive quantitative research based on content analysis
Source: PLoS One. 2024 Mar 7;19(3):e0298601. doi: 10.1371/journal.pone.0298601 (PMC10919601; doi:10.1371/journal.pone.0298601)
Supplement: S1 Data — (ZIP) [file pone.0298601.s001.zip › Data/地级市Z_F工作报告.zip!/║■─╧/╘└╤⌠╩╨/└┤╫╘╣1⁄2╓┌║┼í╛┬φ┐╦╩2╛▌═°í┐_2015.docx]

各位代表：

现在，我代表市人民政府，向大会报告政府工作，请予审议，并请市政协委员和其他列席人员提出意见。

2014年经济社会保持平稳健康发展

2014年，在中共岳阳市委的坚强领导和市人大、市政协的监督支持下，我们认真贯彻落实党中央、国务院和省委、省政府的决策部署，积极应对挑战，奋力攻坚克难，实现了经济社会持续稳步发展，迈上了全面建成小康社会和建设现代幸福岳阳的新台阶。岳阳获评十个“中国最具幸福感城市”、“中国最具文化软实力城市”之一，是全国唯一同获两项殊荣的地级城市。

——经济运行稳中有进。预计全年实现地区生产总值2676亿元，增长9.5%；固定资产投资1827亿元，增长23%；规模工业增加值增长9.5%；社会消费品零售总额884亿元，增长13%；公共财政预算收入256.3亿元，比上年略有增长；完成进出口总额8亿美元，增长24.2%。居民消费价格涨幅控制在2%。新增城镇就业5.7万人，城镇登记失业率4%。

——发展质量稳步提升。三次产业结构调整为10.8∶54.1∶35.1，第三产业比重提高1.1个百分点。高新技术企业达到123家，高新技术产业增加值占GDP比重17%，提高0.3个百分点。非公经济占GDP比重62%，提高0.9个百分点。万元GDP能耗下降6%，主要污染物排放削减率超额完成省定任务，空气质量优良率在全省6个重点监控城市中排第一。连续11年获评《福布斯》中文版中国大陆最佳商业城市。

——居民收入持续增加。民生支出占财政总支出比重72.6%。城乡居民收入分别达23310元和11120元，分别增长10%和12%。城乡居民储蓄存款余额961亿元，增长11.9%。5.8万农村贫困人口实现脱贫。城乡低保月保障标准线分别提高到400元和200元。企业退休人员月人均养老金提高到1604元。

——社会事业蓬勃发展。成功举办全国体育舞蹈公开赛、全省第八届少数民族传统体育运动会。巴陵石化荣获国家科技进步二等奖。中科电气荣获国家技术发明二等奖。巴陵戏首次走出国门赴土库曼斯坦参加对外文化交流，《洞庭渔歌》入选第四批国家级非遗代表名录，成功通过国家历史文化名城复查。人口自然增长率6.5‰，连续16年获评全省人口计生工作先进市，各县市区均进入先进以上行列。获评全国无偿献血先进市、全国卫生监督工作先进市，新农合参合率稳居全省第一。

——社会大局和谐稳定。平安岳阳建设深入推进，安全生产事故各项指标下降10%以上，连续6年获评全国安全生产月活动先进市，连续9年获评全省安全生产工作先进市。信访总量下降11%，进京非访全省最少。社会治安案件发案率下降21.3%，连续8年获评全省社会治安综合治理先进市，综治民调排全省第二位，君山排全省县市区第一位。

过去一年，困难比预料的多，压力比预想的大，但我们顶住压力，克服困难，突出重点，狠抓落实，有力推动了经济社会持续健康发展。

1、突出项目建设，增强发展动力。扎实推进“双百工程”、“三个一批”竞赛活动和“百日攻坚行动”，104个重大建设项目100个开工，103个重大前期项目21个提前开工，南翔万商国际商贸物流城、环南湖旅游交通三圈、科伦二期等项目加快推进，10万吨环氧丙烷等项目竣工投产，国家磁力设备质量监督检验中心建成运营。岳阳港、岳阳三荷机场、常岳九铁路等项目列入国家长江经济带综合立体交通走廊规划，中国洞庭湖博览园、河湖连通工程等项目列入洞庭湖生态经济区“511”工程，1500万吨炼油改扩建及100万吨芳烃项目纳入国家“十三五”石化产业规划布局。绿色化工产业园列入国家首批低碳工业园区试点，军民融合北斗卫星产业园、洞庭湖国际电子商务新城启动建设。汨罗通过国家循环经济标准化试点验收，全省首个农业光伏发电项目落户平江，君山工业园获批省级工业集中区。内联引资到位资金355亿元，华菱集团、红星美凯龙、新华联、友阿等知名企业进驻岳阳。

2、突出改革开放，激发市场活力。行政审批制度改革扎实推进，精简各类行政许可事项166项，在全省率先不再保留非行政许可事项。市、县两级政府机构改革全面启动。工商登记制度改革试点有序开展，新登记企业户数、注册资本(金)分别增长59.6%和98.5%。中信、光大银行岳阳分行开业。铁路运输业、邮政业和电信业“营改增”全面落实。农民住房财产权抵押贷款、农村土地承包经营权确权登记颁证及抵押贷款等试点稳步推进。国企改制遗留问题处理、户籍制度改革进展顺利。临湘列入全国“多规合一”试点市。对外开放迈开大步，“一区一港三口岸”建设取得重大突破，综合保税区是去年全国第一个获批的保税区，汽车整车进口口岸是中部地区首个获批的口岸，进口肉类指定口岸是长江沿线继上海、重庆之后第三个获批的口岸，进口粮食指定口岸正式运营，城陵矶港纳入启运港退税范围，与上港集团成功签订战略合作协议。成功举办“高铁遇上游轮”旅游联盟、央视《寻宝·走进岳阳楼》活动，实现旅游总收入247.4亿元，增长20%。完成集装箱吞吐量22万标箱，港口货物吞吐量1.2亿吨。

3、突出企业帮扶，壮大实体经济。深入开展11大重点产业和37家重点民营企业三年帮扶行动，实施《岳阳市重点民营企业帮扶资金管理办法》。设立高校毕业生创业扶持专项资金，新增银行信贷103亿元，获批建设用地1200公顷，完成各类职业技能培训6万人次。从严整治“三乱、三强、三难”等行为，依法查处破坏经济发展环境典型案件6起。非公经济实现增加值1659亿元，增长10.5%，道道全粮油、中创化工、金龙铜业等6家企业进入湖南民营企业100强。凯美特气获“省长质量奖”，科伦制药、天欣科技获“市长质量奖”。巴陵节能在新三板挂牌。新增中国驰名商标9件、国家地理标志保护产品3件。食品产业成为我市第二个千亿产业。

4、突出城乡统筹，加快小康进程。高速公路“两路一桥”、临湖公路等项目加快推进，芙蓉大道湘阴段竣工通车，新建、改造农村公路504公里，完成危桥改造326座。完成4个110千伏输变电项目建设和419个行政村农网改造。“数字岳阳”地理空间框架建设全面完成。新建高标准基本农田2.2万公顷，疏浚沟渠2万公里，解决农村35.8万人饮水安全问题。粮食总产315万吨，湘阴、华容、岳阳县获评全国粮食生产先进县，汨罗获评全国水稻生产全程机械化示范市和全国平安农机示范市。水产品总量稳居全省第一，湘阴获评全国渔业健康养殖示范县。流转土地309.6万亩，农民合作社发展到2017家。华容获评国家现代农业示范区农业建设与改革试点县，屈原阳岳球获评“全国十佳农民”。王家河流域综合治理、洞庭新城、市体育中心、市档案馆、湖滨和马壕污水处理厂建设加快推进，南湖水上游乐项目投入运营，公共自行车租赁系统使用超过630万人次。深入开展文明创建，城市管理“六大整治”、“八个环境”建设和精神文明“十项重点工作”扎实推进，全省城市卫生暗访检查排名第一。禁拆治违持续加力，拆除违法建设2282处31.7万平米。农村环境整治纵深推进，美丽乡村建设全面启动，平江茅田村获评中国最美休闲乡村。

5、突出民生改善，提升幸福指数。37件省市实事全面或超额完成，开工建设保障性住房44187套，城区提质改造小街巷547条、农贸市场11个，更新新能源公交车200台，实施农村危房改造9700户。“五大保险”继续扩面提标，住房公积金管理规范安全，“零就业”家庭持续动态清零。贫困残疾人住房解困工作扎实推进，在全省率先完成贫困户危房改造“百村示范”工程，获评全国社会扶贫先进集体。教育资源布局调整启动实施，阳光招生扎实推进，城区“大班额”问题化解初见成效，华容纳入全国教育信息化建设试点县。国家公共文化服务体系示范区创建稳步推进，广播电视“村村通”建设任务全面完成，“欢乐潇湘·幸福岳阳”、“周末一元剧场”等群众文化活动深入开展。城镇独生子女父母奖励和单独二孩政策全面落实，完成50家乡镇卫生院改扩建，市二医院、市中医院获批国家级全科医生培训基地，重大疾病防控、公共卫生服务和食品药品安全工作扎实开展。城乡社区规范化、标准化建设水平逐步提高，城区社区工作经费补贴逐年增长，岳阳楼区获评全国和谐社区建设示范城区。大力推进湘江污染防治三年行动计划和河道采砂规范化管理，扎实开展铁山水资源保护行动月活动，加强南湖水环境综合治理，东洞庭湖自然保护区列入首批世界保护地绿色名录。

6、突出民主法治，加强自身建设。自觉接受市人大法律监督和市政协民主监督，全面完成市人大议案、代表建议和市政协建议案、委员提案办理。扎实推进“三提升”主题活动，政风行风明显改进，市政府门户网站荣获中国政务网站领先奖，市长信箱、“12345”热线同获精品栏目奖。乡镇便民服务中心实现全覆盖，政务服务审批提速42.8%。顺利完成第九次村委会换届选举。深入开展党的群众路线教育实践活动，坚决反对“四风”，全面停止新建楼堂馆所，全面完成办公用房清理，全面加强公务用车管理，全市公务卡刷卡率53.3%，“三公”经费支出下降27.9%。

其他各项事业协调发展，国防动员和后备力量建设取得新成绩，国安、武警、消防、人防、统计、史志、保密、老龄、民族宗教、科协、气象、外事侨务、对台、联络接待、移民、烟草、邮政、通信等工作取得新进步，工会、共青团、妇联及各民主党派、工商联、无党派人士在经济社会发展中作出了新贡献。

各位代表!过去一年，奋斗十分艰辛，成绩来之不易，这是省委、省政府和市委正确领导的结果，是市人大、市政协监督支持的结果，是全市上下共同努力的结果。在此，我代表市人民政府，向全市人民，向所有关心和支持岳阳改革发展的社会各界人士，表示衷心的感谢和崇高的敬意!

在总结成绩的同时，我如实向大家报告，受宏观经济下行、长炼和巴陵两厂周期性停产检修、淘汰关停落后产能工业企业等诸多因素影响，去年GDP、规模工业增加值、公共财政预算收入等指标没有达到年初预期。特别是长炼、巴陵两厂近20年来首次全面停产检修70、50天，使全市工业产值和财税收入分别减少92、19.8亿元，拉低GDP、规模工业增加值、公共财政预算收入增速1、2和7.7个百分点，突出反映我市产业结构严重不优，地方工业、非公经济和新兴产业量小质低，转型升级任务艰巨。同时，我们也清醒地看到，经济社会发展中还存在不少困难和问题。主要是：经济稳进向好的基础不牢，重大项目偏少；县域经济综合实力不强，开放型经济薄弱；民生改善仍需加力，社会保障能力和公共服务水平仍需进一步提高；发展环境仍需优化，少数政府公职人员不勤不廉现象仍然存在。对此，我们将采取切实有效措施，认真加以解决。

2015年全力推进湖南通江达海的新增长极建设

今年是全面深化改革的关键之年，是全面推进依法治国的开局之年，是全面建成小康社会的攻坚之年，是全面完成“十二五”规划的收官之年。世界经济仍处在国际金融危机后的深度调整期，缓慢复苏的态势没有改变。我国经济进入发展新常态，总体向好的趋势没有改变。岳阳面临长江经济带和洞庭湖生态经济区两大国家战略叠加的重大机遇，持稳上行的走势没有改变。综合分析判断形势，机遇大于挑战，有利多于不利。特别是省委、省政府提出，要发挥岳阳作为湖南融入长江经济带的“桥头堡”作用，呼应“长三角”，着眼“中三角”，对接长株潭，辐射环湖区，将岳阳发展成为全省通江达海的新增长极。这是省委、省政府对岳阳发展的新定位、新要求，也是岳阳加快发展的新机遇、新动力。我们要以打造“湖南通江达海的新增长极”这一目标来凝聚全市人民的智慧与力量，主动适应新常态，抢抓新机遇，应对新挑战，展现新作为，开创新局面。全力推进“新增长极”建设，必须坚持五条基本路径：建设“桥头堡”，主动融入长江经济带，建设大口岸，促进大流通，培育大产业，构筑开放发展的前沿阵地；呼应“长三角”，全面落实长江流域通关一体化，促进要素合理流动、产业分工协作、市场统一开放，深化与沿江城市合作；着眼“中三角”，发挥岳阳黄金节点区位优势，加强区域内交通、产业、旅游、生态等联动互促，实现抱团竞争与错位发展；对接长株潭，加快推进两型社会滨湖示范区与长株潭城市群产业联动、市场联网、科技联姻和开发联片，基本形成两型发展新模式；辐射环湖区，突出生态文明主题，加强江湖综合治理，推动规划同编、产业同兴、设施同建、环境同治，争当洞庭湖生态经济区建设的先行者、引领者和示范者。

今年政府工作的总体要求是：深入贯彻落实党的十八大及十八届三中、四中全会和中央、省委、市委经济工作会议精神，坚持稳中求进工作总基调，坚持以提高经济发展质量和效益为中心，以全面建成小康社会为总揽，促进“三量齐升”，推进“四化两型”，狠抓改革攻坚，突出创新创业，加快转型升级，强化风险防控，推进依法治市，加强民生保障，努力打造湖南通江达海的新增长极。

今年经济社会发展的主要预期目标是：地区生产总值增长10%左右，规模工业增加值增长10%左右，固定资产投资增长19%，社会消费品零售总额增长12%左右，公共财政预算收入增长10%，城乡居民收入分别增长9%、10%，万元GDP能耗下降3.5%左右。

一、坚持稳增长调结构促转型

科学编制“十三五”规划，在全力稳增长的同时，加大转方式、调结构力度，促进经济提质增效升级，向中高端水平迈进。

扩大有效投资。继续实施重大项目“双百工程”，力争完成全社会固定资产投资2170亿元。交通：加快高速公路“两路一桥”、蒙华铁路岳阳段建设，建成临湖公路、京港澳高速岳阳收费站改扩建等项目，开工建设岳阳三荷机场、京港澳高速新开联络线、G536平江青冲至伍市公路、湘阴武警机场公路等项目，加强农村公路“通畅工程”、危桥改造和渡改桥建设，加大国省干线管养力度，推进常岳九铁路、平益高速前期工作，启动环湖公路规划研究。水利：推进农田水利“百千万”工程、小农水重点县、洞庭湖和黄盖湖综合治理、中小河流治理、水土流失治理和农村小水电站建设，加快市防汛抗旱物资储备中心建设，完成2万公里农村沟渠疏浚和100座病险水库除险加固。电力能源：推进9个110千伏、5个35千伏输变电项目和平江抽水蓄能电站建设，新建改造250千米城乡10千伏线路，启动静脉产业园建设。

发展战略性新兴产业。继续开展重点产业、重点民营企业帮扶行动，推动石化、电力、食品、造纸、电磁、建材、纺织等产业高端化、低碳化、智能化改造，提高传统产业整体竞争力。加快发展北斗应用、生物医药、新材料、节能环保、文化创意等新兴产业，积极培育新模式、新业态、新产品，发掘做大产业新增长点。推动信息化和工业化深度融合，促进信息技术在生产制造、服务业、安全生产等领域的应用。全面推进“135”工程，加快经开区、临港新区、汨罗循环经济产业园等各类园区创新发展，重点培育10家省级以上工业园区，今年力争完成150万平米标准化厂房建设、引进250家创新型企业。

致力打造旅游目的地。加快构建观光、度假、生态、探险、健身等多元旅游产品供给体系，丰富旅游业态，促进旅游融合，推动岳阳旅游由过境地游向目的地游转变。推进中国洞庭湖博览园、南湖十景、顺天洋沙湖国际休闲度假区、岳阳游乐场等20个重大旅游项目建设，加大乡村旅游资源开发，加快休闲度假产业发展。深化高铁、长江沿线旅游城市战略合作，创新举办汨罗江国际龙舟节、君山团湖野生荷花节、洞庭湖国际观鸟节、君山岛爱情文化节等主题节会活动，做强做旺“楼岛江湖”和“花鸟舟情”品牌。完善旅游基础配套，加强旅游市场管理，提升旅游服务品质，真正让游客慕名而来、流连忘返。

促进现代服务业跨越发展。加快建设洞庭湖国际电子商务新城，重点发展食品电商、跨境电商和电商服务，积极培育移动互联网、互联网金融、云计算等新兴业态，全力创建国家电子商务示范城市。加速推进南翔万商国际商贸物流城二、三期和红星美凯龙城市综合体、友阿商业综合体、临港国际汽车城、岳阳茶博城等项目建设，形成功能齐全、结构合理、竞争有序的城市商业网点体系。促进住房消费，推动房地产业平稳发展。大力发展研发设计、服务外包、金融物流、中介咨询等生产性服务业，提速发展健康养老、家政服务、娱乐休闲、体育健身等生活性服务业，培育更多新的经济增长点。

提升文化软实力。充分挖掘历史文化资源，积极培育忧乐文化、求索文化、龙舟文化、爱情文化等特色文化品牌，彰显城市文化魅力，提升城市文化品位。严格实施国家历史文化名城保护规划，切实保护好名城名楼名镇名村名居和巴陵戏、汨罗江畔端午习俗等非物质文化遗产，完成慈氏塔、启动岳阳教会学校等国家级文保单位的维护修缮。发展壮大文化产业，加快屈子文化园、左宗棠文化园等项目建设，举办第二届黄茶文化节，扶持数字出版、移动多媒体、动漫游戏等新兴文化业态，促进会展、文体、影视演映等文化消费，努力打造文化强市。

推进全民创新创业。实施创新驱动发展战略，出台《关于推进创新创业的实施意见》，加快中小企业孵化基地、企业技术研发中心、区域技术创新中心等创新平台建设，加强知识产权保护，大力实施“巴陵人才工程”，建设创新型岳阳。加大中小微企业扶持力度，激活非公经济活力和创造力，促进非公经济健康快速发展。大力弘扬创新创业文化，激发全民创新创业热情，重点打造城镇下岗工人、失地农民、高校毕业生、退役军人、寓外乡友等创业示范园区，举办#from 本文来自学优网http://www.gkstk.com end#首届岳商大会，让创新创业的活力竞相迸发、成果不断涌现。

二、加快“一带一区”开发开放

抢抓长江经济带、洞庭湖生态经济区建设等重大战略机遇，发掘增长新动力，培育开放新优势，实现发展新突破。

优化区域布局。坚持分类指导，发挥比较优势，统筹推进沿江环湖、环长株潭、中心城区三个区域发展。充分发挥华容、临湘、君山、云溪、岳阳县、临港新区等沿江环湖地区通江达海、贯穿东西、政策支持等优势，依托黄金水道，发挥黄金作为。充分发挥湘阴、汨罗、平江、屈原等环长株潭地区毗邻省会城市的优势，实施融长发展战略，承接产业转移，发展“飞地经济”，建设长株潭卫星城市。充分发挥岳阳楼区、经开区、南湖风景区等中心城区商贸、文化、人才、信息、物流等综合优势，重点发展现代服务业和战略性新兴产业，带动全市转型创新发展。

加快港口建设。按照“百万标箱、亿吨大港”的目标，深化城陵矶港与上港集团合作，全力建设湖南通向世界的“出海大通道”。实施长江航道岳阳段疏浚整治工程，改善通航条件，提升航运能力。科学整合港口资源，优化港口功能布局，加快华容塔市驿、君山荆江门、云溪彭家湾、临湘鸭栏、岳阳县鹿角、湘阴漕溪港等港口建设，推进沿江沿湖港口集群发展。切实加强岸线资源保护，严格管理和科学利用岸线资源。

筑牢开放平台。加快“一区一港三口岸”建设，力争综合保税区和汽车整车进口口岸一次性通过验收，推进肉类和粮食进口指定口岸科学运营，大力发展保税加工、保税物流、保税贸易服务，提升开放型经济发展水平。加强沿江口岸通关协作，启动岳阳航运交易所申报，推进岳阳电子口岸、公路口岸建设，争取上海自贸区汽车平行进口政策，建设中部地区功能齐全、通关便利的现代口岸。发挥启运港退税政策优势，力争开通城陵矶至上海洋山港航线，推动进出口贸易扩量倍增。依托“五园一区”平台，开展专业化、产业链、点对点招商，提高招商引资实效。

壮大临港产业。加快推进1500万吨炼油改扩建及100万吨芳烃、15万吨裂化催化剂等项目建设，延伸石化产业链，做大做强绿色化工产业园。扎实推进煤炭储备基地、平江和华容百万千瓦火电厂、小墨山核电站等项目建设，打造沿江绿色能源产业带。大力发展现代物流业，启动中粮城陵矶产业园、华菱欣港物流总部经济项目建设，打造长江中游区域性航运物流中心。积极培育电子信息、食品加工、先进装备制造等优势产业，加快军民融合北斗卫星产业园、洞庭湖绿色食品产业园、新永利专用车等项目建设，构建多点支撑的沿江产业体系。

三、继续全面深化改革

坚定改革信心，狠抓各项改革措施的落地生根，向改革要动力、活力和增长率。

深化经济体制改革。扎实推进新一轮国有企业改革，基本化解国有企业改革历史遗留问题，加快建立现代企业制度，大力发展混合所有制经济。深化投融资体制改革，规范整合政府投融资平台，探索PPP、资产证券化、股权融资等合作模式。加快农村信用社改革，力争完成岳阳农商行组建；引进股份制商业银行，发展资本市场，促进保险证券业和地方小微金融稳健发展。探索科技金融结合的新模式新路径。推进财政收入管理、预算管理、体制管理和绩效管理改革，建立现代财政制度。规范政府性债务管理，防范债务风险。开展政府购买服务试点。落实“营改增”、消费税、资源税、个人所得税等税制改革。启动不动产登记制度改革。加快工商登记制度改革，推进工商注册制度便利化，点燃全民创业的星星之火，形成民营经济蓬勃发展的燎原之势。

深化行政体制改革。继续精简行政审批事项，实施流程再造，纵深推进简政放权、放管结合。加快转变政府职能，全面实行权力清单、负面清单、责任清单制。完成市县政府机构改革。基本完成承担行政职能和从事生产经营活动事业单位改革。深化公务员分类改革，完善基层公务员考录制度。建立最严格的食品药品监管体制。全面实施公车改革。深化行政执法体制改革，探索将县级部分行政执法权依法委托下放到有一定人口规模和经济实力的乡镇。有条件的地方依法启动新一轮并乡(镇)合村试点。

深化社会体制改革。加快教育综合改革，统筹各类教育协调发展，继续推进阳光招生，促进教育公平。全面实施县级公立医院改革和新农合大病商业保险，鼓励支持社会办医，巩固完善基本药物制度和基层运行新机制，深化医疗器械集中采购改革。鼓励产学研协同创新，健全科技成果处置权、收益权管理机制，促进科技成果资本化、产业化。完善文化管理体制，构建现代公共文化服务体系。整合城乡居民基本养老保险、基本医疗保险制度，探索建立城乡一体的社会保险制度。推行阶梯式水、电、气价制度和政府两型采购制度。

深化农村体制改革。全面铺开农村土地承包经营权确权登记颁证，引导土地有序流转，大力培育专业大户、农民合作社、家庭农场、农业企业等新型农业经营主体。加快国有林场改革，完善集体林权制度。推进供销合作社综合改革。加强农村集体资产股份制改革。积极探索农村土地承包经营权抵押贷款。依法稳妥推进农民住房财产权抵押、担保、转让，拓宽农民财产性收入渠道。

四、促进农业稳定发展和农民持续增收

以保障粮食安全和促进农民增收为核心，进一步加强“三农”工作，加快推进农业现代化，巩固和发展农业农村好势头。

夯实农业农村发展基础。扎实推进农村土地综合整治项目建设，确保基本农田总量不减少、用途不改变、质量有提高，确保粮食播种面积稳定在800万亩、总产310万吨以上，保障粮食、生猪、水产等大宗农产品稳定供给。统筹整合涉农资金，确保农业投入只增不减。扩大农业政策性保险，提高农业抗风险能力。大力发展村级集体经济。积极稳妥推进新农村建设，改善人居环境，提高农民素质，推进“物的新农村”和“人的新农村”建设齐头并进。认真抓好减负惠农工作，严格落实涉农补贴政策，关爱农村留守人员，确保农村社会和谐稳定。

加快转变农业发展方式。继续抓好油茶、楠竹、林下经济产业发展，规划建设君山、华容50万亩绿色蔬菜基地，扶持茶叶、油料、饲料等优势产业发展。引导工商资本进入农业，加快岳阳国家农业科技园区、君山全国休闲农业与乡村旅游示范区、岳阳县台湾农民创业园和屈原、华容国家现代农业示范区建设，推进农业生产规模化、标准化、机械化、工厂化、合作化、品牌化。加大农业科技投入，完善农技推广服务体系，提高农业科技水平。积极培育休闲农业、旅游农业、城市农业等新型业态，争创一批休闲农业示范县、示范点和五星级休闲农庄。

加大扶贫攻坚力度。突出革命老区、山区、库区、移民地区等扶贫开发，优化整合扶贫资源，加强驻村扶贫，实施精准扶贫，确保扶贫到村到户。强化产业扶贫、智力扶贫，实施“双千”工程，做好发展生产、公共服务、全民教育三件大事，加快贫困地区脱贫致富步伐。创新扶贫工作机制，引导社会力量参与扶贫事业。坚决打胜扶贫攻坚战，决不让困难群众在全面小康路上落伍掉队。

五、推进新型城镇化

坚持走以人为本、四化同步、优化布局、生态文明、文化传承的新型城镇化道路，努力建设宜居宜业宜游的美丽家园。

坚持规划引领。围绕“一核两带三圈”城市发展战略，编制完成《岳阳市推进新型城镇化实施纲要》，完善城镇规划体系，实现城乡规划全覆盖。优化城镇化布局和形态，依托现有山水脉络，突出滨水生态风格，加快岳阳长江新区建设，推进县城和中心镇扩容提质，严格守住生态红线、耕地红线和水资源红线。强化规划实施全过程监管，持续加大禁拆治违力度，启动城区数字禁违指挥监控平台建设，以铁的措施、铁的纪律，坚决禁住新增违法建设，坚决拆除存量违法建设。

突出产业支撑。坚持城市发展与产业成长“两手抓”，促进城市空间与产业空间高度契合，走以产兴城、以城促产、产城联动、协同共进的发展路子。立足产业特色，坚持优势优先，重点发展岳阳楼区和经开区现代服务业、云溪绿色化工能源、汨罗循环经济、平江食品旅游和汽车部件、湘阴装备制造和食品、岳阳县生物医药和粮食加工、临湘化工建材矿产、华容纺织服装和食品、君山绿色食品和乡村旅游、屈原现代农业等优势产业。依托资源禀赋和区位条件，加快发展镇域经济，宜工则工、宜商则商、宜游则游，重点支持10个不同类型的示范镇建设。

增强城镇功能。坚持地上与地下、硬件与软件、建设与运营相结合，统筹推进城镇水、电、路、气等市政公共设施建设，加大城区旧城改造力度，增加城镇基本公共服务供给，增强城镇承载支撑能力。加快湖滨片区、高铁新城等路网建设，优化城市路网结构；大力发展公共交通，推进岳阳东站客运枢纽、郭镇公交综合基地等站场建设，合理布局建设城市停车场和立体车库，完善道路微循环系统和公共自行车租赁系统，着力缓解城区交通压力。加快马壕污水处理厂建设，强化城镇污染治理设施日常运行监管，推行城市地下管网综合管廊模式。加强文化体育、医疗卫生、就业服务、社保经办和便民服务设施建设，努力构建安全高效便利的公共服务网络体系。

提升管理水平。完善人口管理和服务制度，加快户籍制度改革，加强人口基础信息库建设，全面实施居住证制度，有序推进农业转移人口市民化。坚持集约节约用地，盘活存量用地，强化供地率和用地效益考核。拓宽城镇建设融资渠道，探索民营资本通过特许经营方式进入城镇基础设施建设和公用事业领域，建立多元可持续的资金保障机制。加快智慧城市建设，推进“数字岳阳”地理空间框架成果应用和中心城区平安城市、智慧交通、城市光网建设，提升城市管理数字化、精细化、网格化、规范化水平。继续开展全国环保模范城市、交通模范城市、社会治安综合治理先进市创建工作，巩固提升城市文明度、宜居度、美誉度。

六、建设水墨丹青岳阳

像保护我们的眼睛一样保护生态环境，像呵护我们的母亲一样呵护青山绿水，让岳阳的天空永远蓝下去，让岳阳人民惬意深呼吸。

加快生态岳阳建设。推进“绿色通道”建设三年行动计划，加强森林公园、地质公园、风景名胜区保护，加快城市公园、小游园建设，巩固绿化创模成果，让岳阳城乡郁郁葱葱、鸟语花香。以更大力度推进城区“四湖两河”水域环境治理，加快环南湖旅游交通三圈建设，加强金凤湖生态示范区保护与利用，努力把岳阳打造成为国内外养生养老休闲名城。坚持“两保”并重，加强铁山水资源保护和库区民生保障，全面完成月田、毛田集镇污水处理厂建设，把一泓清甜的生命之源留给子孙后代。实行最严格的水资源管理制度，积极参与长江、湘江、洞庭湖等跨界河湖流域联合治理和开发，抓好东洞庭湖自然保护区建设，加强汨罗江、新墙河等流域联防联治，构建绿色生态走廊。

实施节能减排攻坚。深入推进城区重点排污企业“退二进三”，加快建设环南湖截污管网工程，筹建排污权交易中心，确保污染物排放持续下降。加快淘汰落后产能，推进工业、建筑、交通等重点领域节能，大力发展清洁生产、绿色低碳技术和循环经济。强化大气污染防治，加快燃煤锅炉污染治理设施建设与改造，加强PM2.5监控，加大渣土扬尘、机动车尾气等治理力度，继续淘汰“黄标车”，确保空气质量稳步提高，确保岳阳人民远离“心肺之患”。

深化城乡环境整治。认真落实湘江保护与治理省政府“一号重点工程”，全面完成第一个“三年行动计划”，加强临湘桃林铅锌矿、云溪松阳湖等区域重金属污染治理，确保流域水质持续改善。深入推进“洞庭风雷”行动，严厉打击非法捕捞、采砂、排污、营运等行为，促进人水和谐。继续开展城区“六大整治”行动和爱国卫生运动，全面推进美丽乡村建设，实施农村环境连片整治，让城乡涌现更多美丽县城、美丽乡镇、美丽村庄、美丽社区和文明农户。

七、大力提升人民幸福指数

坚持促发展与惠民生并重，加大民生投入，守住民生底线，不断提高人民生活水平和质量，让岳阳幸福指数更上一层楼。

扎实办好民生实事。全面完成15项24件省级实事：新增城镇就业、农村公路建设、义务教育合格学校和农村公办幼儿园建设、解决农村人口饮水安全、“两房两棚”和“两供两治”建设、0—7岁残疾儿童抢救性康复、贫困重性精神疾病患者救治救助、改扩建精神卫生服务机构、新增社会治安视频监控摄像头、社区矫正中心建设、新增养老服务床位、标准化改造农贸市场、农村环境整治、广播“村村响”、行政村配电网改造。全力办好10件市级实事：完成农村劳动力转移就业3.5万人，完成城区400条小街巷改造，更新100台新能源公交车，建设30个示范社区，建成14所乡镇卫生院，建成市流浪未成年人救助保护中心，建成市老年护理中心，实施重点减排治污项目11个，整治中心城区建筑工地施工噪声扰民，提质改造中心城区56座公厕和18座垃圾站。民生实事桩桩件件关系老百姓切身利益，必须带着责任和感情办，真正把实事办好，把好事办实，办得老百姓满意。

加快发展社会事业。启动新一轮学前教育三年行动计划，推进教育化债，统筹城乡义务教育资源均衡配置，确保人人有学上，力争人人上好学。深入实施重点文化惠民工程，加快市广电中心、青少年活动中心、妇女儿童活动中心、科技馆、图书馆、新墙河抗战文化博物馆建设，完成市档案馆建设，力争成功创建国家公共文化服务体系示范区。推进市中心医院、市妇女儿童医院、市中医院骨伤大楼和5家县级医院建设，推进养老养生健康产业园建设，完善社区卫生服务体系，提高公共卫生服务水平。坚持计划生育基本国策不动摇，稳定适度低生育水平，促进人口长期均衡发展。积极开展全民健身和群众体育活动，提升全民健康水平。

健全完善保障体系。抓好就业这个民生之本，以创新创业带动就业，多渠道增加城乡居民收入。加强价格监测预警，保持物价总水平基本稳定。推进社会保险征缴扩面，出台被征地农民社保政策，继续提高城乡低保、五保供养、医疗救助标准，扩大大病医疗慈善救助基金规模，落实原民办代课教师和老年乡村医生生活困难补助政策，做好下岗职工、大病家庭、贫困残疾人等特殊困难群体帮扶救助工作，构建更加公平可持续的社会保障体系。落实重度残疾人护理补贴制度，促进残疾人康复事业发展。加快市烈士陵园建设。推进殡葬改革，建立普惠型殡葬制度。鼓励和引导民间资本进入养老服务领域，让更多老年朋友安享幸福晚年。

全面加强社会治理。认真贯彻落实新《安全生产法》，加强县乡安全监管能力建设，强化重点领域、重点行业、重点环节安全监管，推进水陆运输超限超载治理工作，坚决防止重特大安全事故发生。深入推进质量兴市和商标战略，切实加强食品药品安全监管，严厉打击制售假冒伪劣行为，营造安全放心的消费环境。深化民间投融资服务机构清理整顿，严厉打击非法集资，防范金融风险。深化平安岳阳建设，加强社会治安防控体系建设，严厉打击各类违法犯罪，巩固综治民调成果，维护社会公平正义。深入开展法治宣传教育，引导全民自觉守法、遇事找法、解决问题靠法，加快建设法治岳阳。深入开展“信访工作双向规范”主题活动，加强人民调解和法律援助，及时就地解决群众合理诉求。加强基层基础工作，全面实施“美好社区、温馨家园”创建活动，推动社区建设管理提质升级。鼓励和支持志愿服务、公益慈善等社会组织发展，激发社会组织活力。加强虚拟社会、突发事件应急管理，完善防灾减灾体系。依法管理宗教事务，推动民族团结进步。加强国防动员、人防、国安、武警、消防等工作，促进军民深度融合发展。

各位代表，做好政府工作，必须加强自身改革建设。要始终以人民满意为出发点和落脚点，加快建设法治政府、效能政府、诚信政府、廉洁政府，不断提高现代治理能力和水平，努力为人民提供优质高效服务。依法行政。认真贯彻党的十八届四中全会精神，把法治理念和法治思维贯穿政府工作全过程，坚持法定职责必须为、法无授权不可为，确保政府工作始终在法治轨道上运行。坚持依法科学决策，建立重大决策合法性审查和风险评估机制，健全法律顾问制度，继续实施专家论证、公开听证和电视问政，自觉接受人大、政协、民主党派和全社会监督。全面推进政务公开，以公开促公正、保公平，确保权力在阳光下运行。提升效能。强化担当精神和效率意识，深入推进“三提升”主题活动，全面加强政府执行力建设，坚决打通决策落地的中梗阻。始终突出问题导向，强化底线思维，以钉钉子的精神狠抓工作落实，划定路线图，制定任务书，确定时间表，压茬推进，精准发力，说了的坚决做到，定了的坚决执行，不搞形式、不放空炮、不走过场。优化环境。认真贯彻落实《湖南省优化经济发展环境十条禁止性规定》，严格执行企业诉求挂号销号、入企检查登记备案和企业宁静日等制度，用硬措施优化软环境，以软环境提升硬实力。践行社会主义核心价值观，加强诚信体系建设，提高全社会文明程度。要用抓项目建设的劲头抓优化环境，要像爱护自己的脸面一样爱护岳阳的形象，积极构建公正、透明、可预期的市场环境，让岳阳成为人人向往的投资创业热土。改进作风。巩固拓展党的群众路线教育实践活动成果，持续深入转作风、反“四风”，既要治理乱作为，也要治理不作为，更要激励公职人员奋发有为，形成作风建设新常态。厉行勤俭节约，精打细算，严管严控，确保“三公”经费支出“零增长”、楼堂馆所“零建设”、一般公务用车“零购置”。严格落实党风廉政建设责任，严守廉洁从政纪律，加强对项目招投标、工程建设、土地出让、政府采购等重点领域和环节的监管，落实制度廉洁性评估，强化行政监察和审计监督，努力营造干净干事、风清气正的良好氛围。

各位代表!550万岳阳人民对美好幸福生活的向往，就是我们矢志不渝的奋斗目标。让我们在中共岳阳市委的坚强领导下，紧紧团结和依靠全市人民，抢抓机遇，开拓创新，扎实工作，为加快全面建成小康社会、打造湖南通江达海的新增长极而努力奋斗!
